# FTY720 Decreases Tumorigenesis in Group 3 Medulloblastoma Patient-Derived Xenografts

**DOI:** 10.1038/s41598-018-25263-5

**Published:** 2018-05-02

**Authors:** Evan F. Garner, Adele P. Williams, Laura L. Stafman, Jamie M. Aye, Elizabeth Mroczek-Musulman, Blake P. Moore, Jerry E. Stewart, Gregory K. Friedman, Elizabeth A. Beierle

**Affiliations:** 1 Division of Pediatric Surgery, Department of Surgery, University of Alabama, Birmingham, Birmingham, AL USA; 2 Department of Pathology, University of Alabama, Birmingham, Birmingham, AL USA; 3 Division of Pediatric Hematology Oncology, Department of Pediatrics, University of Alabama, Birmingham, Birmingham, AL USA

## Abstract

Group 3 tumors account for 28% of medulloblastomas and have the worst prognosis. FTY720, an immunosuppressant currently approved for treatment of multiple sclerosis, has shown antitumor effects in several human cancer cell lines. We hypothesized that treatment with FTY720 (fingolimod) would decrease tumorigenicity in medulloblastoma patient-derived xenografts (PDXs). Three Group 3 medulloblastoma PDXs (D341, D384 and D425) were utilized. Expression of PP2A and its endogenous inhibitors I2PP2A and CIP2A was detected by immunohistochemistry and immunoblotting. PP2A activation was measured via phosphatase activation kit. Cell viability, proliferation, migration and invasion assays were performed after treatment with FTY720. Cell cycle analysis was completed using flow cytometry. A flank model using D425 human medulloblastoma PDX cells was used to assess the *in vivo* effects of FTY720. FTY720 activated PP2A and led to decreased medulloblastoma PDX cell viability, proliferation, migration and invasion and G1 cell cycle arrest in all three PDXs. FTY720 treatment of mice bearing D425 medulloblastoma PDX tumors resulted in a significant decrease in tumor growth compared to vehicle treated animals. FTY720 decreased viability, proliferation, and motility in Group 3 medulloblastoma PDX cells and significantly decreased tumor growth *in vivo*. These results suggest that FTY720 should be investigated further as a potential therapeutic agent for medulloblastoma.

## Introduction

Medulloblastoma is the most common malignant primary nervous system tumor in children^1^. Once considered a singular pathology, medulloblastoma is now more appropriately classified into four molecular subgroups, each having unique clinical and molecular characteristics: Wingless (WNT), Sonic Hedgehog (SHH), Group 3, and Group 4^2–4^. Group 3 tumors account for approximately 25–30% of medulloblastomas and have the worst prognosis^3,5,6^. Despite current multimodal therapy with surgery, chemotherapy and radiation, infants and children with Group 3 tumors have a 5-year overall survival of 45 and 58%, respectively^5^. Furthermore, children who survive often suffer from long-term motor, sensory, endocrine, and neuropsychologic sequelae^7,8^. Novel therapeutic strategies to effectively treat this challenging malignancy are clearly needed.

FTY720 (2-Amino-2-[2-(4-octylphenyl)]-1,3-propanediol, fingolimod) is a synthetic sphingosine immunosuppressant that was approved by the United States Food and Drug Administration (FDA) for the treatment of multiple sclerosis^9,10^. Over the past decade, FTY720 has also been shown to have anti-tumor properties in several human malignancies^11–13^, including glioblastoma^14–16^. FTY720 produces these effects through multiple proposed mechanisms, including activation of the tumor suppressor protein phosphatase 2A (PP2A)^17,18^, down regulation of cyclin D1^19^, inhibition of sphingosine kinase 1 (SphK1)^20,21^, and generation of reactive oxygen species (ROS)^14,22,23^. FTY720 is also thought to induce cell death through caspase-dependent apoptosis^24^ as well as necroptosis^14,25^ and autophagy^14,25^.

Because of the anti-tumor properties of FTY720 seen in other human malignancies, we hypothesized that it may also have anti-tumor effects on medulloblastoma. In this study, we demonstrated that FTY720 treatment led to decreased cell viability, migration and invasion, and caused cell cycle arrest and apoptosis in Group 3 medulloblastoma patient-derived xenografts (PDXs). FTY720 also significantly decreased tumor growth *in vivo* in mice bearing Group 3 medulloblastoma.

## Results

### FTY720 treatment increased PP2A activity

**Figure 1:**
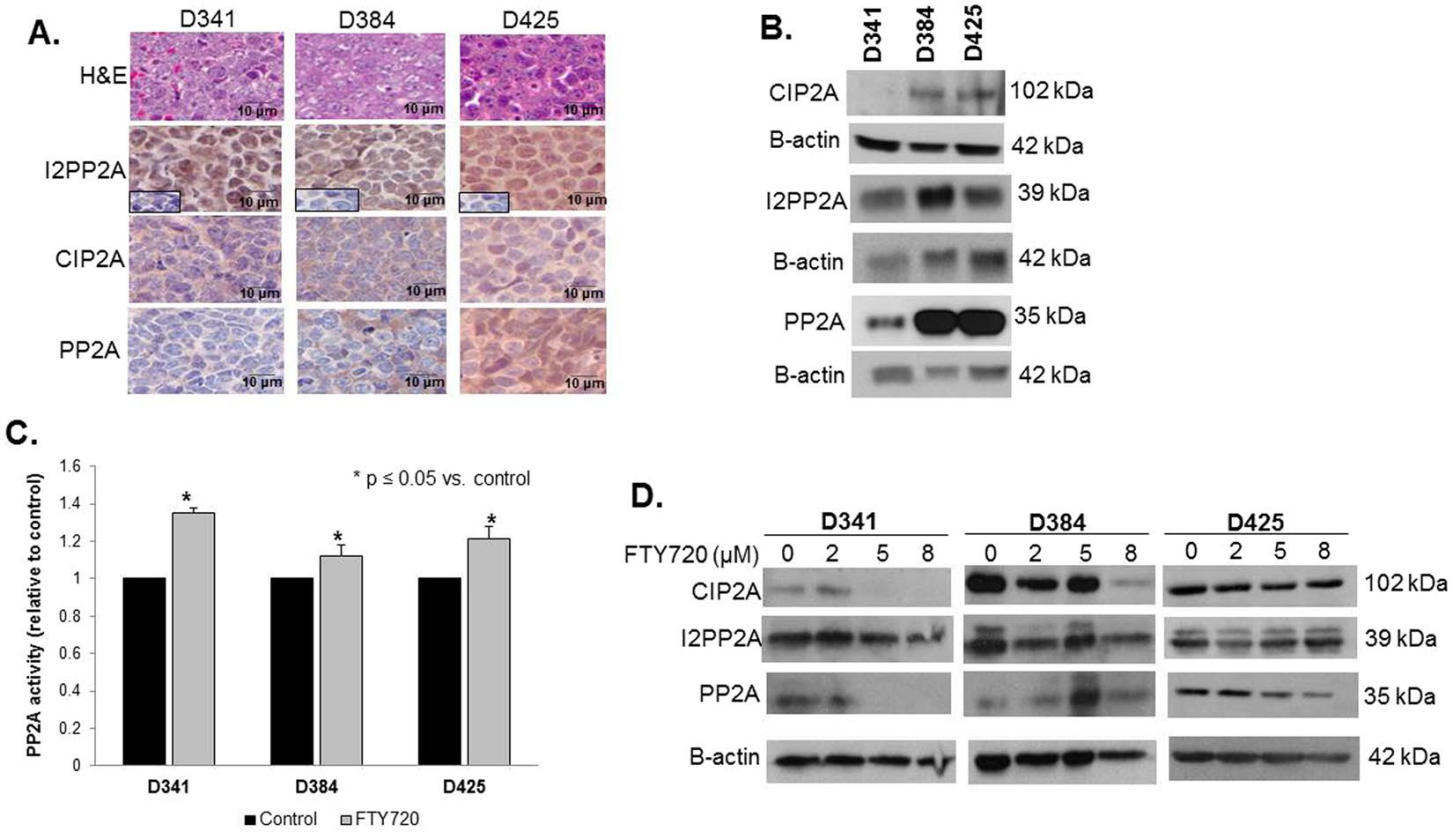
FTY720 treatment increased protein phosphatase 2A activity. (**A**) H&E staining confirmed histology consistent with medulloblastoma in PDXs (*top panels*). Immunohistochemistry was performed to demonstrate I2PP2A, CIP2A and PP2A expression in formalin-fixed, paraffin-embedded human medulloblastoma PDXs. IgG negative controls reacted appropriately (*second row panels, lower left insets)*. (**B**) Immunoblotting confirmed I2PP2A, CIP2A, and PP2A expression in whole cell lysates from these 3 medulloblastoma PDXs. (**C**) PP2A activity was measured in all 3 medulloblastoma PDXs. Treatment with FTY720 (5 µM) for 4 hours resulted in a significant increase in PP2A activity in all three PDX’s tested (*p ≤ 0.05). Experiments were repeated at least in triplicate and reported as mean ± SEM. (**D**) Protein levels of CIP2A and I2PP2A (SET) were measured following FTY720 treatment. CIP2A was diminished in the D341 and D384 MB PDXs (*top left and middle panels*), but not in the D425 MB PDX (*top right panel*). I2PP2A levels were not altered (*middle panels*).

PP2A protein expression has been shown to be downregulated in medulloblastoma tumors and hypothesized to lead to upregulation of tumorigenic signaling pathways^26^. H&E staining confirmed that the PDXs represented medulloblastoma histologically (Fig. 1A, *top panel*). IHC demonstrated that PP2A and its endogenous inhibitors, PP2A inhibitor 2 (I2PP2A/SET) and cancerous inhibitor of PP2A (CIP2A), were present in all three medulloblastoma PDXs (Fig. 1A). Further, immunoblotting confirmed expression of these three proteins in whole cell lysates from the three human Group 3 medulloblastoma PDXs (Fig. 1B). Since activation of the tumor suppressor protein, PP2A, has been proposed as a mechanism by which FTY720 produces its anti-tumor effects^17,18^, the ability of FTY720 to activate PP2A was investigated. Following treatment of D341, D384, and D425 cells with FTY720 (5 µM) for 4 hours, the activity of PP2A was significantly increased over baseline relative to control in all three medulloblastoma PDX cell lines (Fig. 1C). It has been proposed that FTY720 targets the endogenous PP2A inhibitors, I2PP2A/SET and CIP2A, as a mechanism for PP2A activation^27,28^. Protein levels of CIP2A and I2PP2A/SET were measured following FTY720 treatment. CIP2A levels were diminished in the D341 and D384 MB PDXs (Fig. 1D, *top left and middle panels*), but was not changed in the D425 MB PDX (Fig. 1D, *top right panel*). I2PP2A levels were not altered (Fig. 1D, *middle panels*).

### FTY720 prevented progression through the cell cycle in medulloblastoma PDX cells

**Figure 2:**
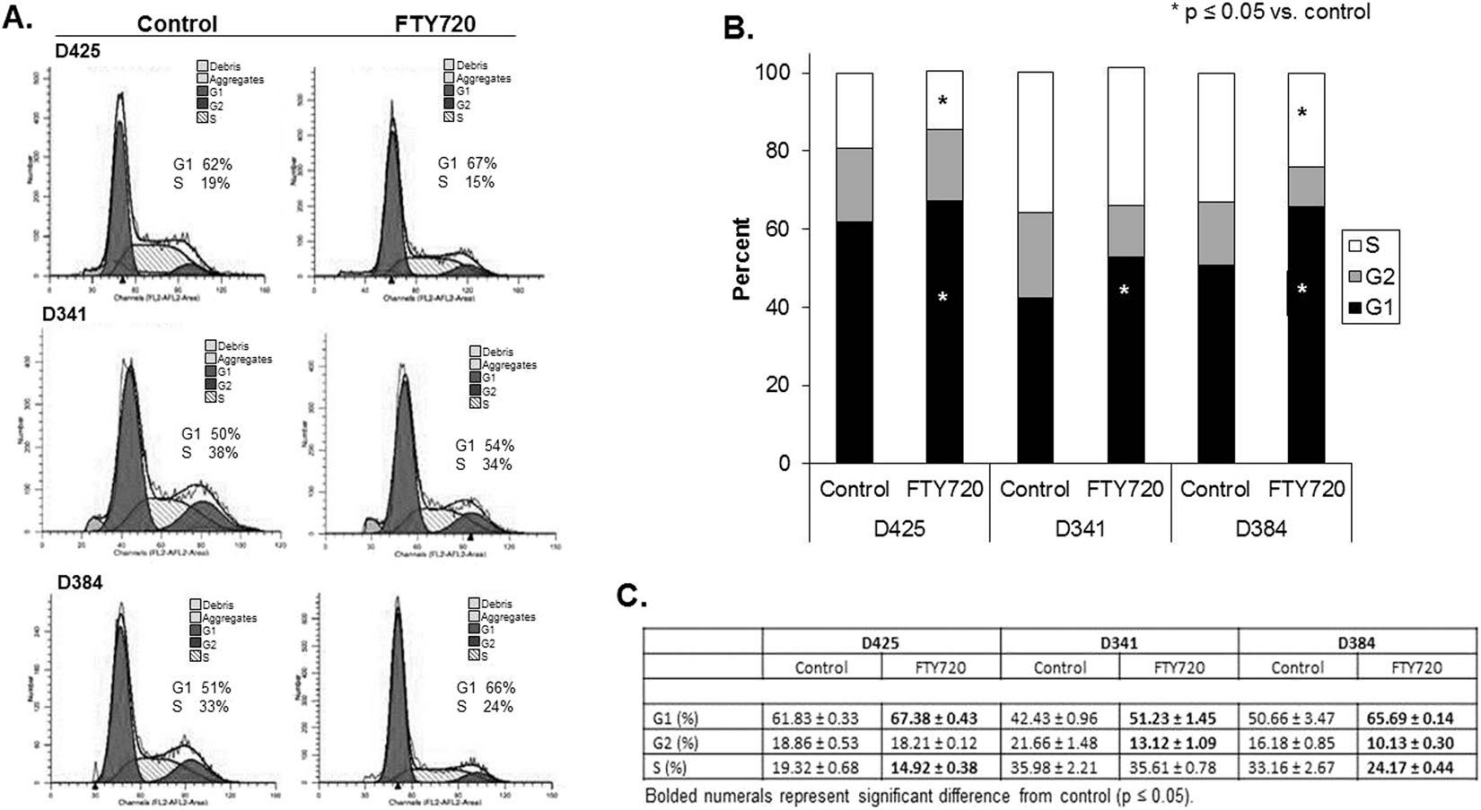
FTY720 treatment of medulloblastoma PDXs led to cell cycle arrest. (**A**) Representative histograms for cell cycle analysis of D425, D341, and D384 human medulloblastoma PDX cells following treatment with FTY720 (5 µM for 24 hours). Cells were analyzed by flow cytometry following staining with propidium iodine. There was an increase in the percentage of cells in the G1 phase and a decrease in the percentage in S phase following FTY720 treatment. (**B**) Graphic representation of cell cycle analysis in D425, D341 and D384 medulloblastoma PDX cells treated with FTY720. There was a significant increase in the G1 phase in cells from all three PDXs (*p ≤ 0.05) and a significant decrease in S phase in the D425 and D384 cells (*p ≤ 0.05) after FTY720 treatment, indicating G1 cell cycle arrest. Experiments were repeated at least in triplicate and reported as mean ± SEM. (**C**) Cell cycle data presented in tabular form reporting mean ± standard error of the mean (SEM). Statistically significant changes are noted with bold type (p ≤ 0.05).

FTY720 is also known to affect cell cycle progression in other cancers^19^, so we hypothesized that medulloblastoma cells treated with FTY720 may fail to progress through the cell cycle. D425, D341 and D384 cells were treated with FTY720 (5 µM) for 24 hours and cell cycle analysis performed using PI staining. Representative histograms from all three PDX cell lines are presented in Fig. 2A. There was a significant increase in the percentage of cells in G1 phase in all three medulloblastoma PDX cell lines (Fig. 2B) indicating G1 cell cycle arrest. In addition, the percentage of cells in S phase in the D425 and D384 PDXs was significantly decreased over baseline controls, further indicating G1 cell cycle arrest (Fig. 2B). S phase in the D341 PDX was also decreased, but did not reach statistical significance. Data are presented in tabular form in Fig. 2C. These data indicate that FTY720 resulted in G1 cell cycle arrest in the medulloblastoma PDXs.

### FTY720 treatment resulted in decreased viability and proliferation and led to apoptosis of medulloblastoma PDX cells

**Figure 3:**
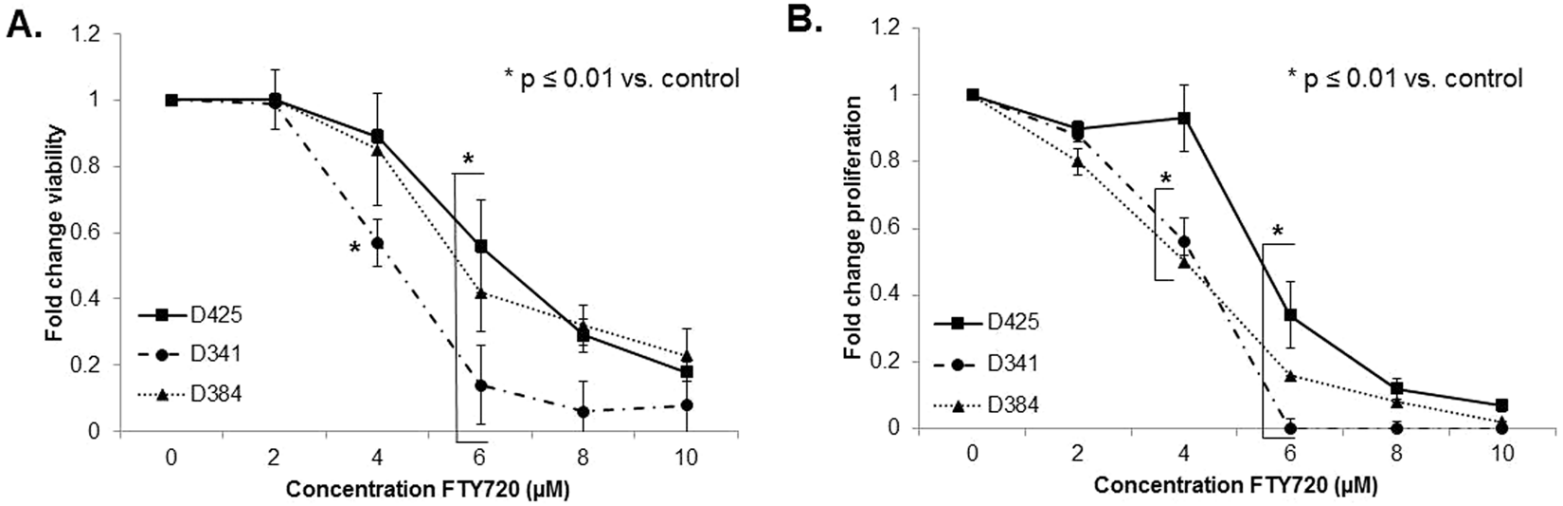
FTY720 treatment resulted in decreased viability and proliferation of medulloblastoma PDXs. (**A**) Cell viability was measured using alamarBlue® assays. D425, D341 and D384 cells were treated with increasing concentrations of FTY720 for 24 hours. Viability was significantly decreased in the D341 beginning at a concentration of 4 µM (*p ≤ 0.01). D425 and D384 cells showed significant decreases in viability at concentrations of 6 µM (*p ≤ 0.01). (**B**) CellTiter 96® assays were used to measure proliferation. D425, D341 and D384 cells were treated with increasing concentrations of FTY720 for 24 hours. Proliferation was significantly decreased in the D341 and D384 cells at 4 µM (*p ≤ 0.01) and at 6 µM (*p ≤ 0.01) in the D425 cells. Experiments were repeated at least in triplicate and reported as mean ± SEM.

FTY720 has been shown to decrease cancer cell viability in a variety of tumor types^11,13,24^, so we examined whether FTY720 would decrease cell viability in medulloblastoma PDXs. Cells were treated with increasing concentrations of FTY720 for 24 hours and viability was measured. FTY720 significantly decreased viability in all 3 medulloblastoma PDX cell lines (Fig. 3A). The lethal dose 50% (LD_50_) for FTY720 was 7.5 µM in D425, 5.5 µM in D341, and 7.3 µM in D384 cells. Cell proliferation was also measured following treatment for 24 hours with increasing concentrations of FTY720. There was a significant decrease in proliferation seen in all 3 medulloblastoma PDXs following FTY720 treatment (Fig. 3B).

**Figure 4:**
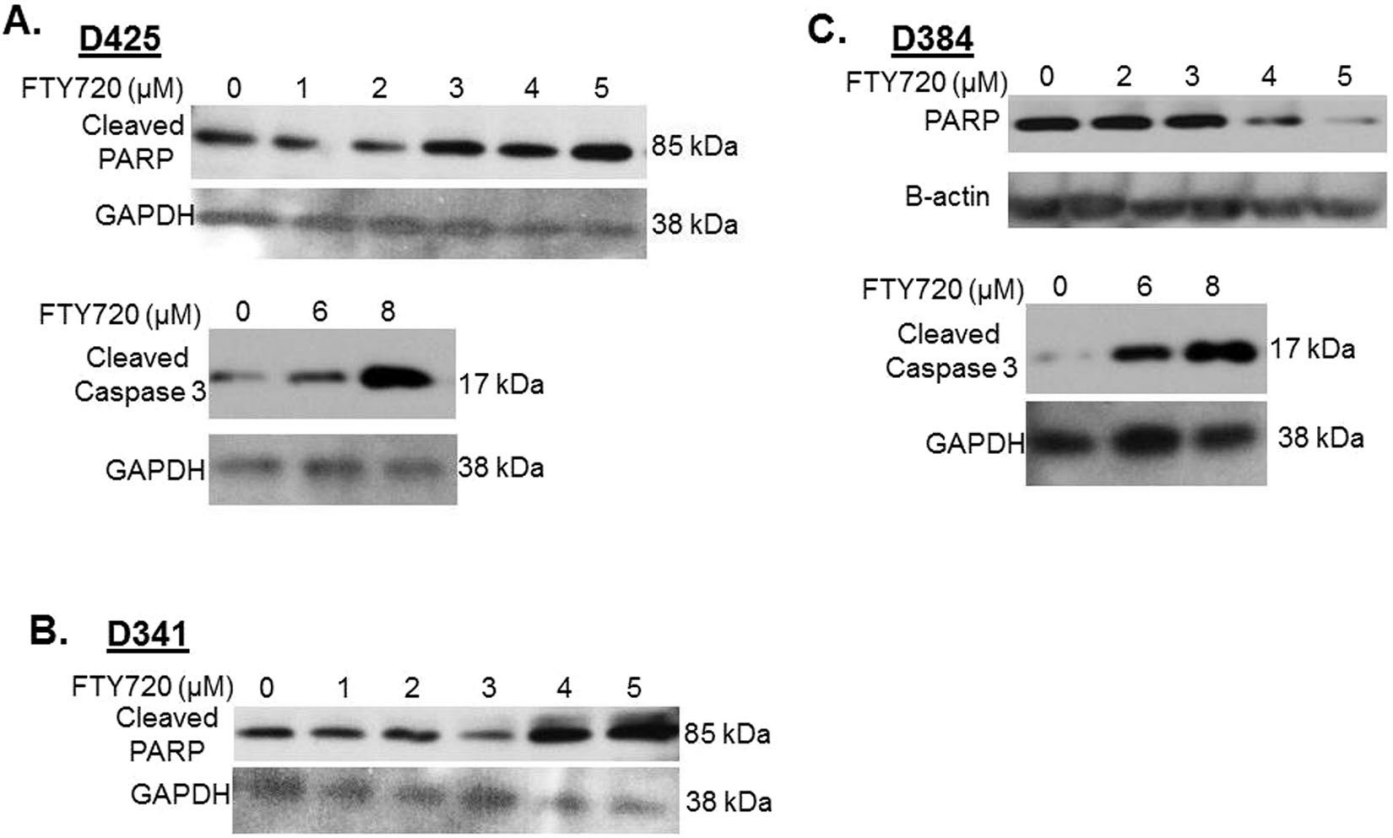
FTY720 treatment resulted in apoptosis of medulloblastoma PDX cells. Western blotting for cleaved PARP, total PARP, and cleaved caspase 3 of whole cell lysates from medulloblastoma PDX cells treated with increasing concentrations of FTY720. (**A**) In D425 cells, FTY720 resulted in increased cleaved PARP and increased caspase 3 indicating apoptosis. (**B**) In D341 cells, increasing concentrations of FTY720 led to increased cleaved PARP, again indicating apoptosis. (**C**) D384 cells, treated with increasing concentrations of FTY720, showed decreased total PARP and increased cleaved caspase 3, both indicating apoptosis. Β-actin or GAPDH was utilized to demonstrate equal protein loading.

Since FTY720 treatment induced cancer cell apoptosis in other cancers^16,22,23^, we next determined whether the decrease in cell viability observed with FTY720 in medulloblastoma PDX cells may be secondary to apoptosis. Apoptosis was measured by immunoblotting for either loss of total PARP, or an increase in cleaved PARP or an increase in cleaved caspase 3, all of which are known to be indicative of apoptosis. In the D425 PDX, there was an increase in both cleaved PARP and cleaved caspase 3 with increasing concentrations of FTY720 (Fig. 4A, *upper and lower panels*). Cleaved PARP was also increased in the D341 cell line with FTY720 treatment (Fig. 4B). In the D384 cells, total PARP decreased (Fig. 4C, *upper panels*) and cleaved caspase 3 increased (Fig. 4C, *lower panels*) with increasing concentrations of FTY720. These data showed that FTY720 treatment resulted in apoptosis in the medulloblastoma PDX cells.

### Migration and invasion of medulloblastoma PDX cells was decreased after treatment with FTY720

**Figure 5:**
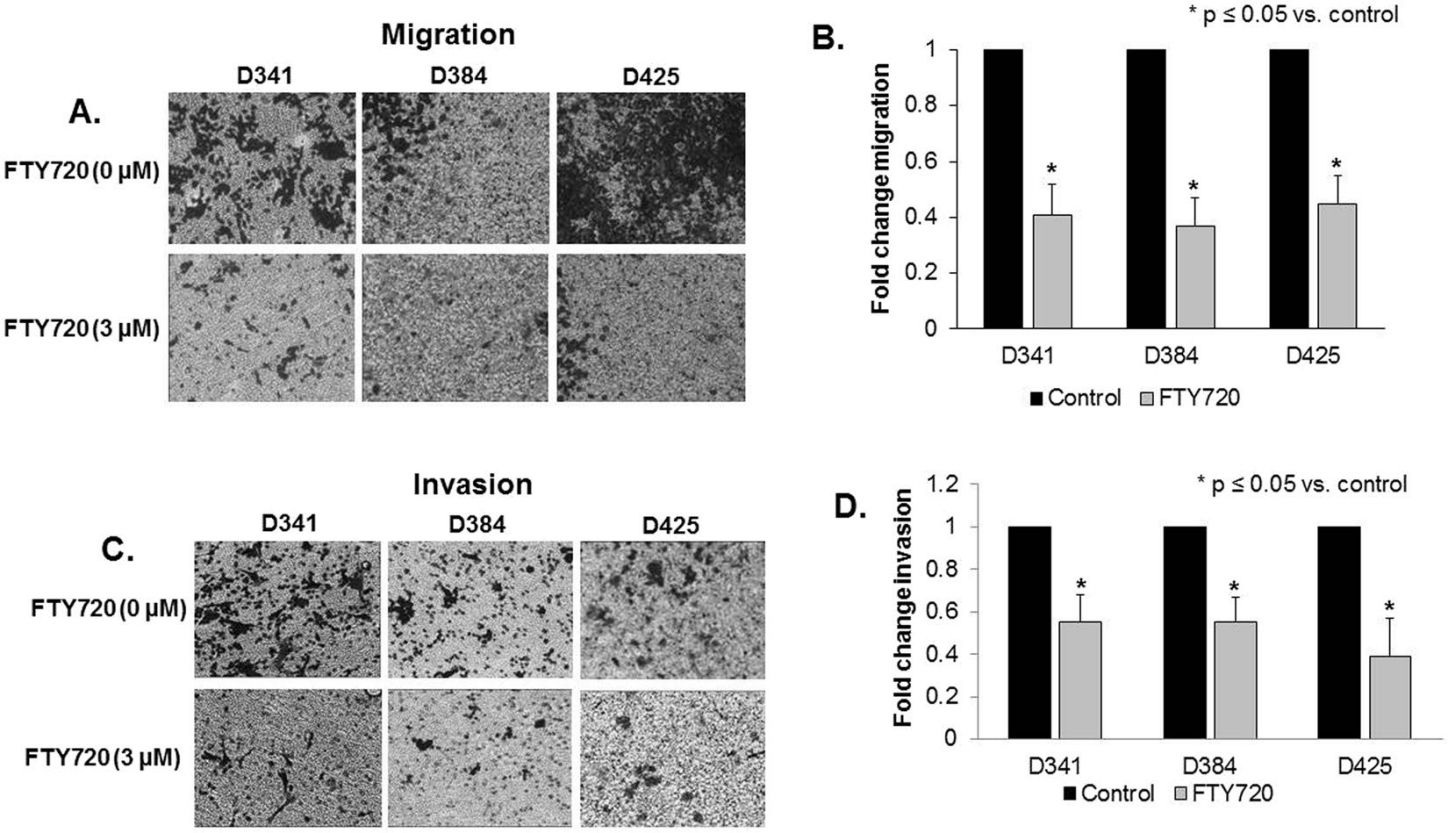
Migration and invasion of medulloblastoma PDX cells was decreased after treatment with FTY720. (**A**) Migration was determined using Transwell® inserts with 8 μM pores, coated on the bottom with laminin. Cells were treated with FTY720 (0, 3 µM) for 24 hours and allowed to migrate for 24 hours. Representative photographs of the inserts show decreased cell migration in all 3 PDXs with FTY720 treatment. (**B**) The number of cells migrating was quantitated and reported in graphic form, again showing over 50% decrease in migration in all 3 PDXs after treatment with FTY720. (**C**) Invasion was completed using Transwell® inserts with 8 μM pores, coated on the bottom with laminin and the inside of the inserts coated with Matrigel™. Cells were treated with FTY720 (0, 3 µM) for 24 hours and allowed to invade for 24 hours. Representative photographs of the inserts show decreased cell invasion in all 3 PDXs with FTY720 treatment. (**D**) The number of cells invading was quantitated and reported in graphic form, demonstrating significantly decreased invasion in all 3 PDXs after treatment with FTY720. Photographs are representative of three independent experiments showing similar results. Graphs are means of three experiments with data reported as mean ± SEM.

The ability to migrate and invade is a hallmark behavior of cancer cells. Therefore, we investigated whether FTY720 treatment decreased medulloblastoma cell migration and invasion. Since non-viable cells will not migrate or invade, cells were treated with concentrations of FTY720 (0 or 3 µM) well below their LD_50_ and migration and invasion was assessed after 24 hours. FTY720 resulted in a significant decrease in both migration (Fig. 5A,B) and invasion (Fig. 5C,D) in all 3 medulloblastoma PDXs.

### FTY720 decreased medulloblastoma tumor growth *in vivo*

**Figure 6:**
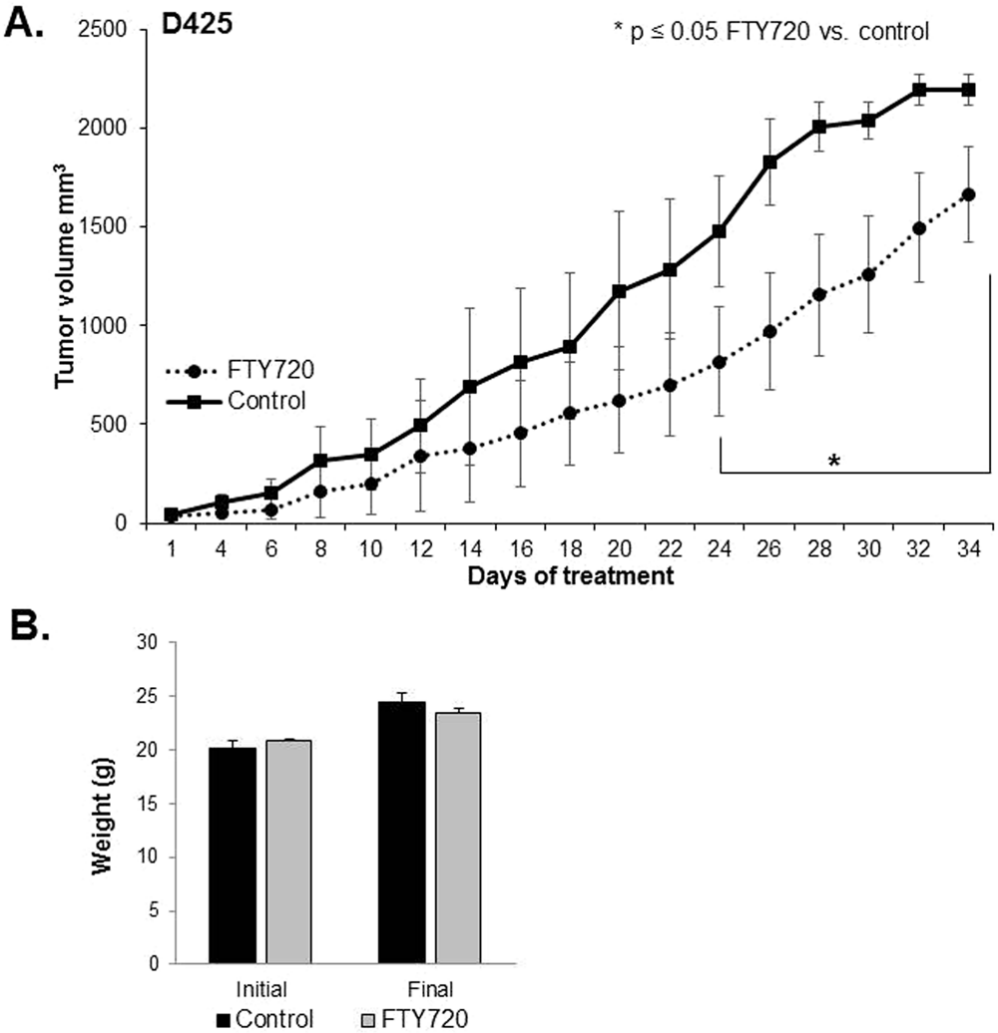
FTY720 decreased medulloblastoma tumor growth *in vivo*. (**A**) D425 cells (2.5 × 10^6^ cells in 25% Matrigel™) were injected into the right flank of 6-week-old, female, athymic nude mice. When tumors reached an average of 100 mm^3^, mice were randomized to receive either vehicle (N = 7) or FTY720 (10 mg/kg/day) (N = 8) for 5 weeks. Animals treated with FTY720 (*closed circles*) had significantly smaller tumors than those treated with vehicle alone (*closed squares*). (**B**) Mice were weighed at the beginning of the experiment and at the time of euthanasia. There was no significant difference in animal weights between those treated with vehicle and those treated with FTY720.

For *in vivo* testing of FTY720 against medulloblastoma tumor growth, D425 human medulloblastoma PDX cells (2.5 × 10^6^ in Matrigel™) were injected into the right flank of athymic nude mice (n = 15). The flank model was chosen as it allowed us to accurately measure changes in tumor size over time. Once tumors were palpable (100 mm^3^) animals were randomized to receive daily oral doses of vehicle (N = 7) or FTY720 (10 mg/kg/day) (N = 8) for 5 weeks. This dosage was chosen based on previous literature reports^15,29,30^. This time period was based upon prior experiments in our laboratory showing that 5 weeks was the usual maximal survival for animals with untreated tumors. At the end of 5 weeks of treatment, all surviving animals were euthanized. The animals treated with FTY720 showed a significant decrease in tumor volume when compared to vehicle treated animals (Fig. 6A). At 5 weeks, there were no surviving animals in the vehicle treated group and 3 in the FTY720 treated group. The individual tumor growth curves were also markedly different, with the vehicle-treated tumors doubling in size twice as fast as the FTY720 treated tumors (Supplemental Data Fig. 1). The median time for tumor doubling in the vehicle treated group was 4 days (range: 2–8 days) compared to 8 days (range: 5–13 days) in the FTY720 treated animals. FTY720 did not significantly affect the weight of the animals (Fig. 6B).

## Discussion

Despite aggressive therapy including surgery, multi-agent chemotherapy and craniospinal radiation, outcomes for children with Group 3 medulloblastoma remain poor, and survivors often suffer from debilitating life-long toxicities related to these interventions. There is a great need for new therapies to improve outcomes and reduce the toxicities from current treatments. Most current investigations are focused on discovery of new compounds, the repurposing of previously approved compounds, and the development of novel combinatorial therapies.

The use of immunosuppressant drugs to fight cancer is not a new concept and is one that has been implemented with a number of compounds^31,32^. FTY720, a metabolite from *Isaria sinclairii*, was developed as an immunosuppressant for use in transplantation, and is currently utilized in the treatment of multiple sclerosis. Recently, FTY720 was found to have an inhibitory effect upon human cancer cells. In 2001, Sonoda and colleagues reported that FTY720 induced apoptosis in human glioma cells^33^ and another group reported FTY720 treatment led to apoptosis of glioma stem cells^16^. Subsequent studies showed similar effects with decreased viability and apoptosis in other human tumor types including bladder cancer^11^, hepatoma^34^, pancreatic cancer^24^ and leukemia^35^. These promising findings prompted an investigation of the potential impact of FTY720 on medulloblastoma.

The mechanism of action of FTY720 in cancer cells is not completely defined. It has been hypothesized that FTY720 activates PP2A^17,18^ thereby leading to tumor cell death. FTY720-induced PP2A activation has been postulated to occur through direct effects or through targeting of I2PP2A/SET^27^ or CIP2A^28^, the endogenous inhibitors of PP2A. PP2A is a known tumor suppressor that is inactive or downregulated in many human tumors either through upregulation of its endogenous inhibitors, I2PP2A/SET^36,37^ or CIP2A^38–40^, or through mutations^41^ which limit the activity of PP2A. PP2A has been shown to be downregulated in medulloblastoma tumors leading to upregulation of tumorigenic signaling pathways^26^. In the current study, we demonstrated that PP2A and its two endogenous inhibitors I2PP2A/SET and CIP2A are expressed in medulloblastoma PDX cells. In addition, FTY720 treatment of human medulloblastoma PDXs resulted in activation of PP2A in the face of decreased overall PP2A protein expression, but changes in I2PP2A/SET or CIP2A expression were not consistently seen. These findings have been reported by other authors who have noted that CIP2A or I2PP2A/SET expression was not altered by FTY720^42^.

In this study, treatment of human medulloblastoma PDX cells with FTY720 arrested the cell cycle at G1. These effects were noted with low concentrations of FTY720 (5 µM). Other investigators have demonstrated similar findings. Permpongkosol *et al*. showed an increased percentage of DU145 prostate cancer cells in G1 and decreased percentage in S phase following FTY720, but at a much higher dose (20 µM)^43^. Increased percentages of cells in G1 were seen with FTY720 treatment of both gastric cancer cells^44^ and in acute myeloid leukemia cells^45^ in concentrations similar to those utilized in the current study (5 µM).

In addition to reducing cell viability, FTY720 has been shown to reduce tumor cell migration and invasion. Zhang and others showed that human glioma cells had a marked reduction in migration and invasion following FTY720 treatment^15^. Another study in human prostate cancer cells demonstrated a decrease in migration and invasion with FTY720 treatment^46^. Similarly, in the current study, FTY720 in doses well below the LD_50_ resulted in significant reductions in both migration and invasion in human medulloblastoma cells. Further, as postulated by previous investigators^15^, the AKT pathway may be involved in these findings as AKT phosphorylation was markedly decreased in D341 and D384 cells with increasing concentrations of FTY720 (Supplemental Fig. 2). FTY720-induced dephosphorylation of AKT has been noted in other tumor types including mesothelioma^47^, prostate cancer^48^ and breast cancer^49^.

Other investigators have proposed changes in ERK phosphorylation as a potential downstream target of FTY720. Rincon and colleagues showed decreased activation of ERK in BT-474 and MDA-MB-231 breast cancer cells following treatment with FTY720^49^. Cristóbal and colleagues reported similar results with PC-3 prostate cancer cells. When these cells were exposed to 10 µM FTY720, ERK phosphorylation was decreased^48^. In the current study, D384 medulloblastoma PDX cells showed decreased ERK phosphorylation without a change in total ERK expression following FTY720 treatment (Supplemental Data Fig. 2). On the contrary, phosphorylation of ERK was increased in the D341 PDX cells with increasing doses of FTY720. These data indicate that the effects of FTY720 on kinases may be cell line dependent, and along with the findings for AKT, will serve as a basis for further mechanistic studies of FTY720 effects in medulloblastoma.

Using an *in vivo* mouse xenograft model of D425 human medulloblastoma, we found that FTY720 treatment led to a significant decrease in tumor growth compared to untreated animals. A flank model was chosen to allow more careful determination of tumor growth over the time period of study and to adhere to the three R’s of animal use; reduce, replace, and refine. We believe that FTY720 would be applicable to the orthotopic location as it has been documented to pass the blood brain barrier and have effects upon intracranial glioblastoma^16^ as well as other intracranial pathologies^50,51^.

Our results provide evidence that FTY720 decreased medulloblastoma cell viability, migration, invasion, and *in vivo* tumor growth. These findings are novel as FTY720 has not yet been studied as a therapeutic tool for medulloblastoma. Furthermore, the use of human PDX medulloblastoma cell lines in these studies is also important as it provided a clinically relevant model for the preclinical study of this compound in medulloblastoma. These data suggest that FTY720 may be a novel therapeutic for treating medulloblastoma and further clinical evaluation is warranted.

## Materials and Methods

### Patient-derived medulloblastoma xenografts

Three Group 3 medulloblastoma xenografts^52,53^ established from pediatric patients were used for experiments: D341 Med (D341), D384 Med (D384), and D425 Med (D425). These tumors were generously provided by Darell D. Bigner, MD, PhD, Duke Medical Center^29,30^. The xenografts were maintained in athymic nude mice (Envigo, Pratville, AL). After tumors were harvested, the cells were dissociated using a Tumor Dissociation Kit (Miltenyi Biotec, San Diego, CA) per manufacturer’s protocol. All cell lines were maintained in neurobasal medium (Life Technologies, Carlsbad, CA) supplemented with B-27 supplement without Vitamin A (Life Technologies), N2 supplement (Life Technologies), amphotericin B (250 μg/mL), gentamicin (50 μg/mL), *L*-glutamine (2 mM), epidermal growth factor (10 ng/mL; Miltenyi Biotec) and fibroblast growth factor (10 ng/mL; Miltenyi Biotec). The cells were kept under standard conditions at 37 °C and 5% CO_2_. All three medulloblastoma PDXs were verified within the last 12 months using short tandem repeat analysis (Heflin Center for Genomic Sciences, UAB, Birmingham, AL).

### Reagents and antibodies

FTY720 was purchased from Cayman Chemical (10006292, Cayman Chemical, Ann Arbor, MI). Primary antibodies used for Western blotting included the following: anti-I2PP2A (H-120) (sc-25564) from Santa Cruz Biotechnology (Santa Cruz, CA), anti-PP2A (ab32104) and anti-CIP2A (ab99518) from Abcam (Cambridge, MA), anti-total AKT (9272), anti-phospho-AKT (S473; 9271), p44/42 MAP Kinase [ERK1/2 (9102)], anti-phospho-p44/42 MAPK [phospho-ERK, T202/T204, (4377)], anti-total PARP (9542) and anti-cleaved Caspase-3 (9661) from Cell Signaling Technology (Danvers, MA), anti-β-actin from Sigma (A1978, Sigma Aldrich, St. Louis, MO), anti-cleaved PARP (MAB3565) and anti-GAPDH (MAB374, clone 6C5) from EMD Millipore (EMD Millipore, Billerica, MA).

### Immunohistochemistry

Formalin-fixed paraffin-embedded xenograft tumor specimens were sectioned into 6 µm sections and baked at 70 °C for one hour on positive slides. Slides were deparaffinized, steamed, quenched with 3% hydrogen peroxide, and blocked with blocking buffer [bovine serum albumin (BSA), powdered milk, Triton X-100, phosphate buffered saline (PBS)] for 30 minutes at 4 °C. The primary antibodies anti-I2PP2A/SET (rabbit polyclonal, 1:400, sc-25564, Santa Cruz), anti-CIP2A (rabbit polyclonal, 1:200, ab99518, Abcam), and anti-PP2A (rabbit monoclonal, 1:200, ab32104, Abcam) were added and incubated for 1 hour in a humidity chamber at room temperature. After washing with PBS, the secondary antibody for rabbit (R.T.U. biotinylated universal antibody, Vector Laboratories, Burlingame, CA) was added for 1 hour at 22 °C. The staining reaction was developed with VECTASTAIN Elite ABC reagent (PK-7100, Vector Laboratories) and Metal Enhanced DAB Substrate (Thermo Fisher Scientific). Slides were counterstained with hematoxylin. Negative controls (rabbit IgG, 1 µg/mL, EMD Millipore) were included with each run. Routine hematoxylin and eosin staining was performed on each specimen.

### PP2A activity assay

Cells (1 × 10^6^ cells) were treated with FTY720 (5 µM) for 4 hours and then lysed using NP-40 lysis buffer. PP2A activity was measured using a PP2A Immunoprecipitation Phosphatase Assay Kit (17–313, EMD Millipore). This kit measures activity of the C subunit of PP2A (clone 106). Briefly, protein lysates were incubated with PP2A antibody at 4C with continuous rotation for 2 hours. Following the addition of assay buffers and malachite green solution, the plate was read at an absorbance of 650nm using a microplate reader (Epoch Microplate Spectrophotometer, BioTek Instruments, Winooski, VT). Phosphatase activity was determined using a standard curve. Experiments were repeated at least in triplicate and phosphatase activity was reported as mean fold change ± standard error of the mean (SEM) from the untreated sample for each cell line.

### Immunoblotting

Briefly, cells were lysed on ice for 30 minutes in a buffer consisting of 50 mM Tris-HCl (pH 7.4), 150 mM NaCl, 1 mM EDTA, 1% Triton x-100, 1% sodium deoxcycholate, 0.1% SDS, phosphatase inhibitor (P5726, Sigma Aldrich), protease inhibitor (P8340, Sigma Aldrich), and phenylmethylsulfonyl fluoride (PMSF, P7626, Sigma Aldrich). The lysates were then centrifuged at 14 000 rpm for 30 minutes at 4 °C. Protein concentrations were determined using a Micro BCA™ Protein Assay Kit (Thermo Fisher Scientific, Rockford, IL), separated by electrophoresis on SDS-PAGE gels, and transferred to Immobilon®-P polyvinylidene fluoride (PVDF) transfer membrane (EMB Millipore). Precision Plus Protein Kaleidoscope Standards (161-0375, Bio-Rad, Hercules, CA) were used for molecular weight markers to confirm expected size of target proteins. Antibodies were used in accordance with the manufacturers’ recommended protocol. Samples were visualized by enhanced chemiluminescence (ECL) using Luminata Classico and Luminata Crescendo Western horseradish peroxidase (HPR) substrates (EMD Millipore). Anti-β-actin was used an internal control to ensure equal protein loading between samples.

### Cell cycle analysis

Cell cycle analysis was performed using propidium iodine (PI) staining and flow cytometric evaluation. Cells (1 × 10^6^ cells) were plated and treated with FTY720 (5 µM). After 24 hours, a single cell suspension was achieved using Accutase® (Sigma). The cells were then washed with PBS and fixed in 1mL of ice-cold, 100% ethanol overnight. The cells underwent a second PBS wash and were stained for 1 hour with 200 μL of staining solution consisting of 20 μg/mL propidium iodide [(PI), Invitrogen, Carlsbad, CA], 0.1% Triton X (Active Motif, Carlsbad, CA) and RNAse A (0.1 mg/mL, Qiagen, Valencia, CA). The samples were analyzed with fluorescence-activated cell sorting (FACS) using a FACSCalibur™ Flow Cytometer (BD Biosciences, San Jose, CA). ModFit LT software (Verity Software House Inc., Topsham, ME) was used to analyze the data.

### Cell viability and proliferation assays

An alamarBlue® assay (Thermo Fisher Scientific) was performed to assess cell viability following treatment with FTY720. Cells (1.5 × 10^3^ cells) were plated and treated with increasing concentrations of FTY720. After 24 hours, 10 μL of alamarBlue® dye was added to each well. The plates were read at using a microplate reader (Epoch Microplate Spectrophotometer) to record the absorbance at 570 nm, using 600 nm as a reference wavelength. Experiments were completed in triplicate and viability reported as fold change ± SEM.

Proliferation was assessed using the CellTiter 96® Aqueous One Solution Cell Proliferation assay (Promega, Madison, WI). Medulloblastoma cells (5 × 10^3^ cells) were plated and treated with FTY720 at increasing concentrations. After 24 hours, 10 μL CellTiter96® dye was added to each well and the absorbance was measured at 490 nm using a microplate reader (Epoch Microplate Spectrophotometer). Experiments were repeated in triplicate and proliferation reported as fold change ± SEM.

### Cell migration and invasion assays

Cell migration and invasion assays were performed using 6.5 mm Transwell® inserts with 8 μM pore polycarbonate membrane (Corning Inc., Corning, NY) in 24-well culture plates. The bottoms of the inserts were coated with laminin (10 μg/mL). For invasion assays, the inside of the inserts were also coated with Matrigel™ (1 mg/mL, 50 µL; BD Biosciences) overnight at 37 °C and then washed with PBS. Cells were pretreated with FTY720 (0, 3 µM) for 24 hours and then 1.5 × 10^5^ cells plated into the top of the insert. The insert was then placed into a well containing 300 μL of treated media containing 10% fetal bovine serum (FBS) as a chemo-attractant. After 24 hours, the cells on top of the inserts were removed using a cotton swab. The inserts were then fixed in 3% paraformaldehyde prior to staining with crystal violet. The imaging software SPOT Basic 5.2 (Diagnostic Instruments Inc., Sterling Heights, MI) was used to take pictures of the inserts at predetermined locations with a microscope at 100× and then the cells were quantified using ImageJ software (Ver 1.49, available online at http://imagej.nih.gov/ij)^54^. Experiments were repeated in triplicate and migration and invasion reported as fold change ± SEM.

### Animal statement

Animal experiments were approved by the University of Alabama, Birmingham Institutional Animal Care and Use Committee (IACUC-09355) and were conducted within institutional, national, and NIH guidelines.

### *In vivo* tumor growth

D425 cells (2.5 × 10^6^ cells in 25% Matrigel™; Corning Inc.) were injected into the right flank of 6-week-old, female, athymic nude mice (Envigo, Pratville, AL). Once tumors were palpable (100 mm^3^), the mice were randomized to receive either 50 µL suspension vehicle (ORA-Plus®, Perrigo, Allegan, MI) or FTY720 10 mg/kg/day suspended in 50 µL ORA-Plus® once daily via oral gavage for 5 weeks. The FTY720 dosing was based on previous animal studies^16,47,55^. The flank tumors were measured twice weekly using calipers and tumor volume was calculated using the formula [(width^2^ × length/2] with length being the largest measurement. The mice remaining at the end of the treatment period were humanely euthanized.

### Statistical analyses

Experiments were performed at a minimum of triplicate. Densitometry was performed utilizing Scion Image Program (http://www.nist.gov/lispix/imlab/prelim/dnld.html). Each band was normalized to background, then to their respective β-actin, and finally the phospho band was normalized to the total protein expression band. All bands were then normalized to 0 µM treatment group as previously reported^56^. Data reported as the mean ± standard error of the mean. Data between groups was compared using an analysis of variance or Student’s *t* test as appropriate. Statistical significance was defined as p < 0.05.

### Data availability statement

No data sets were generated in the current studies.

## Electronic supplementary material

Supplementary information
