# Supplementary material for: FTY720 Decreases Tumorigenesis in Group 3 Medulloblastoma Patient-Derived Xenografts
Source: Sci Rep. 2018 May 2;8:6913. doi: 10.1038/s41598-018-25263-5 (PMC5932040; doi:10.1038/s41598-018-25263-5)

FTY720 Decreases Tumorigenesis in Group 3 Medulloblastoma Patient-Derived Xenografts

Evan F. Garner[1], Adele P. Williams[1], Laura L. Stafman[1], Jamie M. Aye[2], Elizabeth Mroczek-Musulman[2], Blake P. Moore[3], Jerry E. Stewart[1], Gregory K. Friedman[3], Elizabeth A. Beierle[1*]

SUPPLEMENTARY INFORMATION

Figure 1. FTY720 decreased medulloblastoma tumor growth *in vivo*. D425 cells (2.5 x 10^6 cells in 25% Matrigel™) were injected into the right flank of 6-week-old, female, athymic nude mice. When tumors reached an average of 100 mm^3, mice were randomized to receive either vehicle (N = 7) or FTY720 (10 mg/kg/day) (N = 8) for 5 weeks. Animals treated with FTY720 had significantly longer time until tumor size doubled (*right graph*) compared to those treated with vehicle (*left graph*).

Figure 2. FTY720 led to decreased AKT and ERK phosphorylation in whole cell lysates from medulloblastoma human PDXs. Western blotting of whole cell lysates from D341 and D384 PDX cells treated with increasing concentrations of FTY720 demonstrated decreased phosphorylation of AKT and ERK without corresponding changed in total AKT or total ERK. Densitometry confirmed increased changes noted.

Supplemental data
Fig. 1

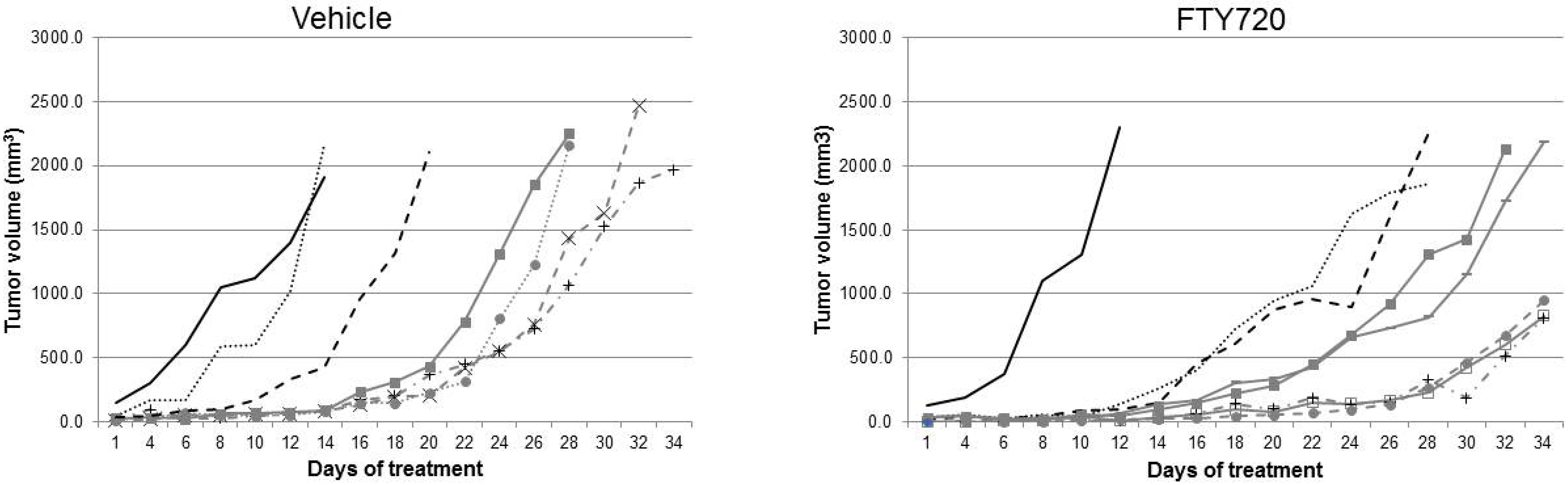

Supplemental data
Fig. 2

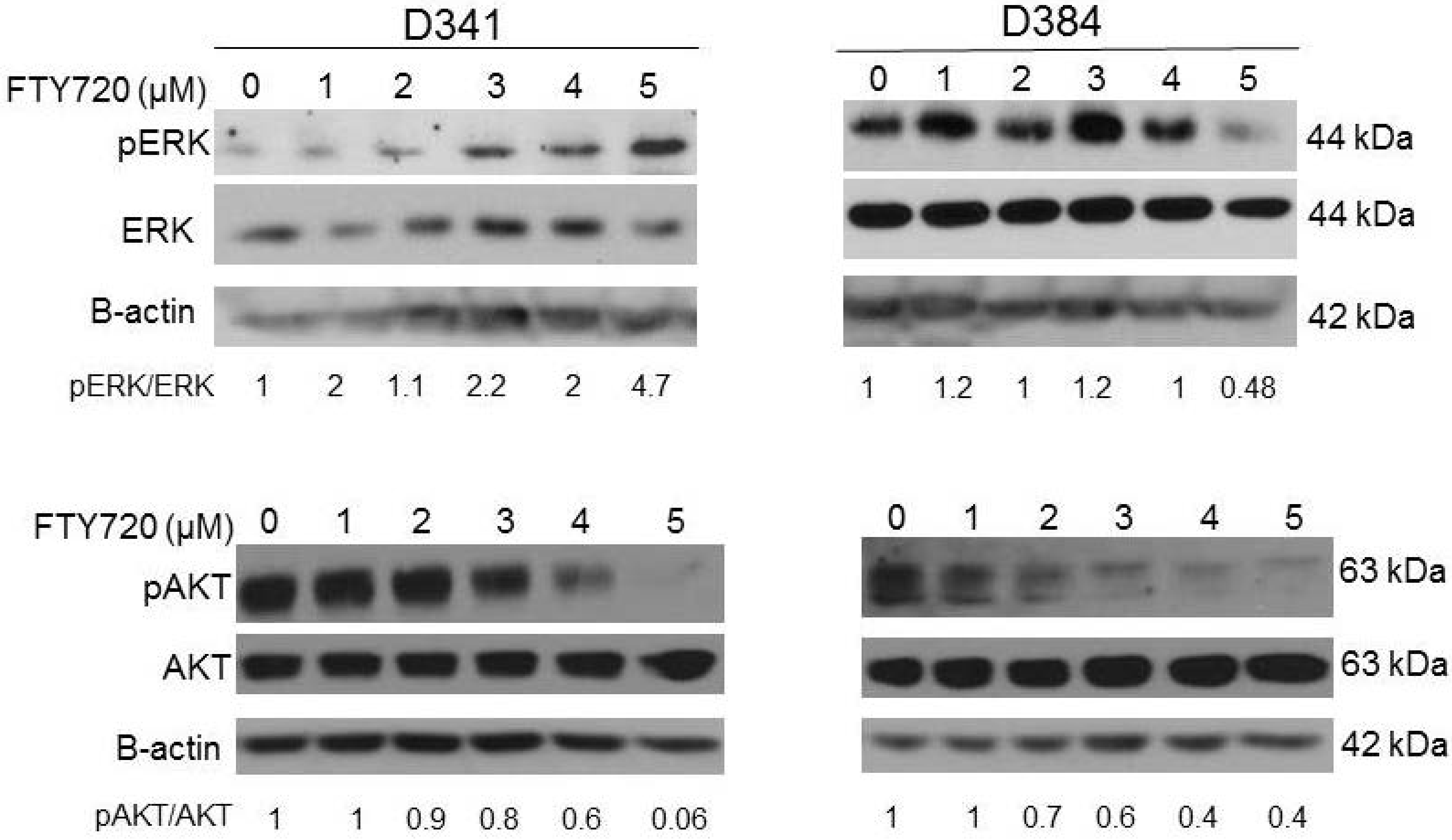

Supplement: Supplementary file 1 — Supplementary information [file 41598_2018_25263_MOESM1_ESM.pdf]
